# Serum D-dimer is a potential predictor for thromboembolism complications in patients with renal biopsy

**DOI:** 10.1038/s41598-017-05210-6

**Published:** 2017-07-06

**Authors:** Xia Tan, Guochun Chen, Yu Liu, Letian Zhou, Liyu He, Di Liu, Yexin Liu, Fan Zhang, Huiqiong Li, Hong Liu

**Affiliations:** Department of Nephrology, The Second Xiangya Hospital of Central South University. Renal Research Institute of Central South University, Key Lab of Kidney Disease and Blood Purification in Hunan, Changsha, China

## Abstract

Renal biopsy has been widely recommended in clinic to determine the histological patterns of kidney disease. To prevent bleeding complications, patients should routinely stop anticoagulants prior to renal biopsy. However, patients with kidney disease are susceptible to thromboembolisms, particularly in those with severe hypoalbuminemia. This study was designed to investigate the application of serum D-dimer as a predictor for thrombotic events after renal biopsy. 400 consecutive native renal biopsies were prospectively included in this 2-month follow-up study. The overall incidence of bleeding and thrombotic complication is 4%, including hematuria or large perinephric hematoma (2.5%, n = 10) and thrombotic complication (1.5%, n = 6). Compared to low serum D-dimer (<2.00 μg/ml), subjects in the group of high serum D-dimer (≥2.00 μg/ml) were more incline to develop thrombotic complications (9.1% versus 0.3%; RR, 30.33; p < 0.001). D-dimer correlated positively with age (r_s_ = 0.258, P < 0.001). Inverse correlations were found for albumin (r_s_ = −0.339, P < 0.001). Taken together, patients with high serum D-dimer carry an increased risk of thrombotic complications after renal biopsy. Our findings suggest that serum D-dimer can serve as a potential predictor for thrombotic events in patients with kidney disease. Further cautions should be given to these subjects.

## Introduction

Percutaneous renal biopsy is an important diagnostic tool to obtain a histological diagnosis for specific patient treatment. Some studies have been done on renal biopsies to examine risk factors for biopsy complications^1–5^ Hemorrhage is the most common complication. Previous studies always took gross hematuria, perinephric hematoma as minor complications, bleeding requiring transfusion, a hemodynamically significant arteriovenous fistula, any radiological or surgical intervention and death as major complications. But rarely study reported thrombotic complications. Patients with kidney disease especially nephrotic syndrome can become hypercoagulable. In these patients, venous thromboembolism ranging from 2% in children^6^ to as high as 26.7% in adults^7^ and a relative risk of arterial thromboembolism ranging from 1 to 5.5 have been reported^8, 9^. The pathophysiological mechanisms of thromboembolism in patients with nephrotic syndrome have yet to be unraveled. Nevertheless, alterations in plasma levels of antithrombin III, protein C, and protein S, enhanced platelet aggregation, hyperviscosity, and hyperlipidemia, as well as treatment with corticosteroids and diuretics, are considered predisposing factors for the development of thrombotic events. Before and after renal biopsy should stop anticoagulants. The procedure also could activate coagulation system. At our institution, some patients admitted serious thrombotic complications after renal biopsy. It is important to select the patients who at high risk of thrombotic complication if taken renal biopsy.

D-dimer is a degradation product of cross-linked frbrin which exists in the blood after a thrombus is degraded by thrombin, coagulation factor XIIIa and fibrinolysin. D-dimer levels can reflect the activity of coagulation and the fibrinolytic system. Previous studies have demonstrated that expression levels of D-dimer in serum are significantly increased following thromblic diseases^10–16^. However, to our knowledge, no study has examined if D-dimer prior renal biopsy can also be used to predict thrombotic complications after renal biopsies.

## Methods

In this study, 400 consecutive native renal biopsies (200 in men and 200 in women) were prospectively included (Table 1). The excluded criterions were as follow: patients with uncontrolled hypertension (systolic blood pressure ≥150 mmHg at the time before procedure); patients requiring ongoing anticoagulation; patients with significant liver disease (including known cirrhosis) or platelet counts of <100 × 10^9/L. Patients were told to stop any anticoagulants, nonsteroidal anti-inflammatory drugs and anti-platelet agents at least 3 days prior to the biopsy. Data were collected from our centre from May 1, 2016, until September 30, 2016. All of these patients were followed 2 months after renal biopsies.

**Table 1:** Baseline Data of All Kidney Biopsies.

	Kidney Biopsies, n = 400	
	Male = 200	Female = 200
	Median (Range)	Mean (SD)
Age, years	40 (14–75)	40.2 (15.4)
Serum creatinine, μmol/L	77.5 (31.7–1297.4)	123.6 (138.3)
eGFR MDRD, mL/min per 1.73 m^2^	85.79 (4.81–226.80)	85.22 (41.96)
Needle size, gauge	16	
D-dimer, μg/ml	0.45 (0.03–9.94)	1.00 (1.43)
Thrombotic comlication, n (%)	6 (1.5)	
Bleeding or large hematoma complications, n (%)	10 (2.5)	

The study was approved by the “Medical Ethics Review Committee of The Second Xiangya Hospital of Central South University”. Informed consent was obtained from all participants. All methods were performed in accordance with the guidelines and regulations of the National Natural Science Foundation of China and the Medical Ethics Review Committee of The Second Xiangya Hospital of Central South University.

All biopsies were performed with the use of real-time ultrasound guidance and an automated spring-loaded biopsy device. The size of the needles used were 16-gauge (Tochigi-Shi. Tochigi-Ken, Japan).

Clinical data including sex, age, systolic and diastolic blood pressure, weight, coagulation function, protein C, protein S and albumin were collected. D-dimer levels were measured before renal biopsy with ELISA method (Table 2). Kidney function was determined by means of estimated glomerular filtration rate (eGFR, Modification of Diet in Renal Disease [MDRD]).

**Table 2:** Demographic Data of Patients With D-dimer <2.00 μg/ml and ≥2.00 μg/ml.

	D-dimer <2.00 μg/ml, n = 345		D-dimer ≥2.00 μg/ml, n = 55	
	Male = 171, Female = 174		Male = 29, Femal = 26	
	Median (Range)	Mean (SD)	Median (Range)	Mean (SD)
Age, years	37 (14–73)	39 (15)	53 (15–75)	50 (16)
Serum crentinine, μmol/L	76.3 (31.7–1297.4)	111.73 (124.90)	101.4 (45–703.1)	193.87 (188.41)
eGFR MDRD, mL/min per 1.73 m^2^	88.70 (4.81–226.80)	88.66 (40.47)	57.70 (7.03–173.66)	63.67 (45.01)
Thrombotic comlication, n (%)	1 (0.3)		5 (9.1)	
Bleeding or large hematoma complications, n (%)	9 (2.6)		1 (1.8)	

After kidney biopsy, we followed these patients 2 months. Adverse effects after the biopsy procedure were divided into bleeding (bleeding requiring blood transfusion or invasive procedure, a hemodynamically significant arteriovenous fistula, any radiological or surgical internention and death) or large hematoma complications and thrombotic complications (e.g., deep venous thrombosis, pulmonary embolism, acute myocardial infarction) that diagnosed by clinical manifestations, imaging and laboratory test results.

For statistical analysis, the IBM SPSS Statistic 19 was used. Fisher’s exact test and χ^2^ analyses were used for the cross-tabulation of data. Correlation analyses were performed by use of Spearman’s test (r_s_). Multiple regression analyses were performed by use of Stepwise analysis. Binary logistic regression analysis was used. Data are presented as risk ratios (RR) and confidence intervals (CI). The Mann-Whitney U test was used. A 2-side P value of <0.05 was considered significant.

### Data availability

The datasets analysed during the current study are available from the corresponding author on reasonable request.

## Results

The overall hematuria or large perinephric hematoma complication and thrombotic complication rate was 4% (n = 16), including bleeding or large hematoma (2.5%, n = 10) and thrombotic (1.5%, n = 6) complications. In the group of bleeding or large hematoma complications, nine patients had clinically significant hematoma (extended hospital care and treatment for pain) and one patients had extended hospital care for over half a month because of gross hematuria (prolonged observation, intravenous fluid replacement). In the group of thrombotic complication group, two patients had pulmonary embolism (PE) in the first week after renal biopsy, two patients had acute myocardial infarction in the first month after renal biopsy, one patient had deep venous thrombosis (DVT) of right lower limb, one had mesenteric thrombosis.

**Figure 1:**
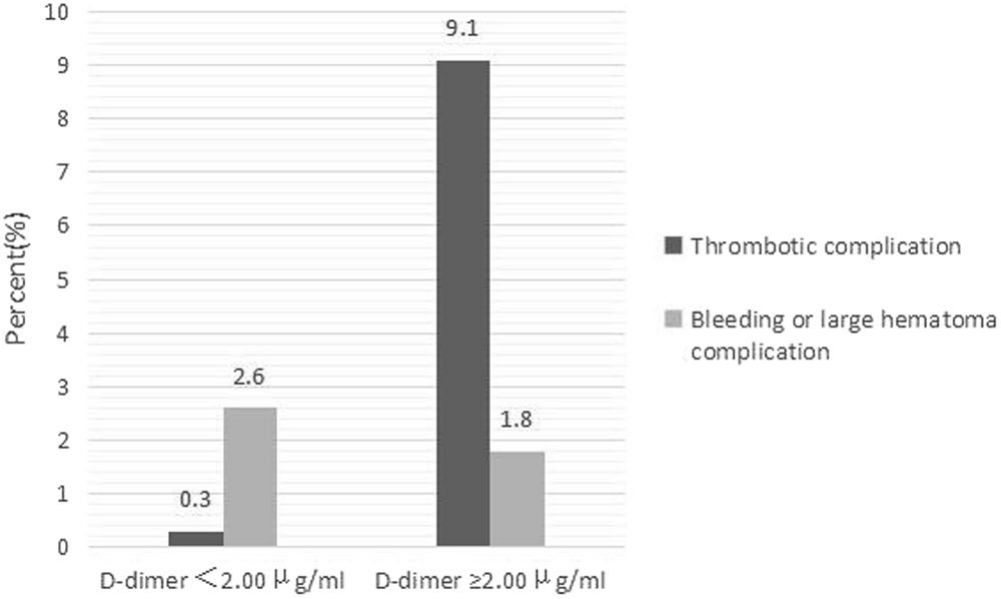
D-dimer and thrombotic or bleeding complications.

The median and mean of D-dimer level was 0.45 mg/l and 1.00 mg/l. There was no difference in D-dimer level between sexes. There was a cutoff-level for D-dimer as a predictor for thrombotic complications after renal biopsy 2 months, when D-dimer was ≥2.00 μg/ml versus <2.00 μg/ml. This resulted in different thrombotic complications (9.1% versus 0.3%; RR, 30.33; p < 0.001) (Fig. 1). Binary logistic regression analysis was performed, inclucing complications (bleeding or large hematoma complications and thrombotic complications) as dependent factor and as variables age, sex, systolic and diastolic blood pressure, weight, albumin, and D-dimer ≥2.00 μg/ml and <2.00 μg/ml. The significant variable in this model was D-dimer ≥2.00 μg/ml (P < 0.001) and systolic pressure <120 mmHg (P = 0.043) for thrombotic complications. It did not demonstrate a statistical difference for bleeding or large hematoma complications.

**Figure 2:**
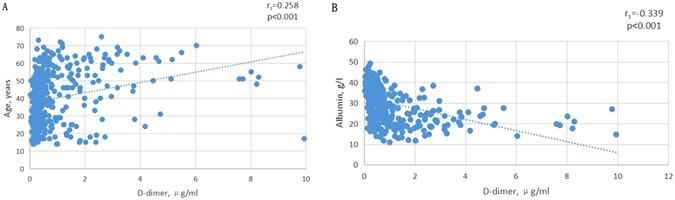
Scatter diagram for the correlation of D-dimer with age and albumin. (**A**) Positive correlation of D-dimer level with age. (**B**) Negative correlation of D-dimer level with albumin.

To assess what affected the D-dimer, we investigated for correlations to various variables. In the linear regression analysis, D-dimer correlated positively with age (r_s_ = 0.258, P < 0.001). Inverse correlations were found for albumin (r_s_ = −0.339, P < 0.001) (Fig. 2). No correlations were existed between D-dimer and sex, systolic and diastolic blood pressure, weight and eGFR.

**Figure 3:**
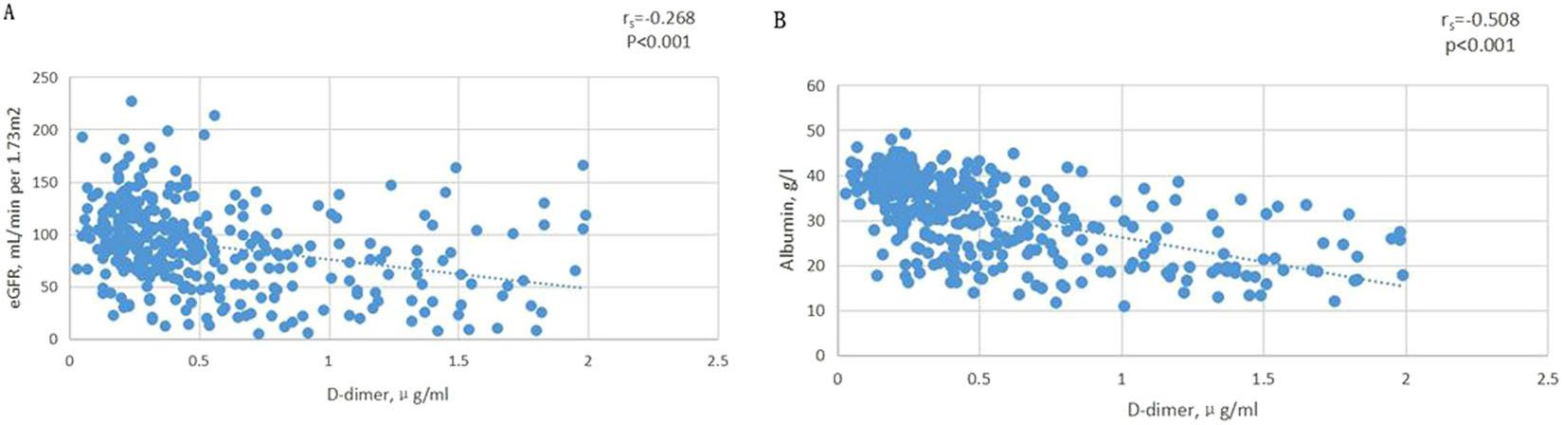
Scatter diagram for the correlation of D-dimer with eGFR and albumin in D-dimer <2.00 μg/ml. (**A**) Negative correlation of D-dimer level with eGFR. (**B**) Negative correlation of D-dimer level with albumin.

A sub-analysis was performed for D-dimer levels of <2.00 μg/ml and ≥2.00 μg/ml. In the group D-dimer <2.00 μg/ml, D-dimer correlated negatively with eGFR (r_s_ = −0.268, P < 0.001) and albumin (r_s_ = −0.508, P < 0.001) (Fig. 3) but not with systolic and diastolic blood pressure, age, sex and weight. In the group ≥2.00 μg/ml, D-dimer had no correlation with systolic and diastolic blood pressure, age, sex, weight albumin, and eGFR.

Multiple regression stepwise analyses were performed with the D-dimer as the dependent factor and the independent variables ages, systolic and diastolic blood pressurem weight, albumin and eGFR. When including all D-dimer, the model was significant (r^2^ = 0.153, P < 0.001) with significant variables age (P < 0.001) and albumin (P < 0.001). When subgroup analyses were performed for D-dimer<2.00 μg/ml, the model’s r^2^ was 0.310 (P < 0.001), with eGFR (P < 0.001), sex (P = 0.037), albumin (P < 0.001) as the significant variables. For D-dimer ≥2.00 μg/ml, the model was not significant.

The coagulation function was analysed (Table 3). There were no differences in prothrombin time (PT), activated partial thromboplastin time (APTT), antithrombin III (AT-III) levels between D-dimer levels <2.00 μg/ml and ≥2.00 μg/ml groups. The protein C (P = 0.015) and fibrin degradation product (FDP) (P < 0.001) were higher in D-dimer levels ≥2.00 μg/ml group than D-dimer <2.00 μg/ml group (Table 4). The AT-III (P = 0.012) was higher in D-dimer <2.00 μg/ml group compared with D-dimer levels ≥2.00 μg/ml group.

**Table 3:** Comparison of the Prothrombin time (PT), Activated partial thromboplastin time (APTT), Antithrombin III (AT-III) and Fibrin degradation product (FDP) of Patients With different D-dimer levels.

	D-dimer <2.00 μg/ml, n = 345	D-dimer ≥2.00 μg/ml, n = 55	P value
	Male = 171, Female = 174	Male = 29, Femal = 26	
	Mean (SD)	Mean (SD)	
PT, sec	11.97 (1.05)	11.99 (1.53)	0.930
APTT, sec	35.48 (5.93)	34.88 (7.06)	0.556
AT-III, %	101.16 (20.42)	93.27 (20.45)	0.012
FDP, μg/ml	2.73 (1.60)	13.33 (7.95)	<0.001

**Table 4:** Comparison of the Protein C and Protein S of Patients With D-dimer <2.00 μg/ml and ≥2.00 μg/ml.

	D-dimer <2.00 μg/ml, n = 345	D-dimer ≥2.00 μg/ml, n = 55	P value
	Male = 171, Female = 174	Male = 29, Femal = 26	
	Mean (SD)	Mean (SD)	
Protein C, %	133.00 (44.12)	150.71 (57.61)	0.015
Protein S, %	98.81 (25.75)	107.34 (29.15)	0.054

One of the patient didn’t achieve sufficient tissue for diagnosis. The mean of D-dimer was more than 2.00 μg/ml in crescentic nephritis and lupus nephritis (II–V). The D-dimer was higher in crescentic nephritis (4.04 versus 0.96, P < 0.001) and lupus nephritis (II–V) (3.25 versus 0.95, P < 0.001) compared with other diagnoses (Table 5).

**Table 5:** Distribution of Histological Diagnoses, Thrombotic or Bleeding complications, and D-dimer.

Histological Diagnosis	Kidney Biopsies, n = 399%	Bleeding or large hematoma complications, n = 10	Thrombotic complications, n = 6	D-dimer (Mean)
Primary				
IgA nephritis	28.57 (114)	1.75 (2)	0.88 (1)	0.48
Membranous nephropathy	21.05 (84)	1.19 (1)	3.57 (3)	1.49
MCN	12.03 (48)	2.08 (1)	2.08 (1)	0.69
FSGS	14.79 (59)	1.69 (1)	1.69 (1)	0.65
Mesangioproliferative glomerulonephritis	3.01 (12)	8.33 (1)	0	0.84
Crescentic glomerulonephritis	1.75 (7)	0	0	4.04
Diffuse hyperplasia nephritis	1.00 (4)	0	0	1.13
membranoproliferative glomerulonephritis	0.25 (1)	0	0	3.84
Proliferative sclerosing glomerulonephritis	1.50 (6)	0	0	1.15
Sclerosing glomerulonephritis	0.50 (2)	0	0	0.56
Secondary				
Diabetic nephropathy	3.01 (12)	8.33 (1)	0	1.37
Lupus Nephritis	2.76 (11)	9.09 (1)	0	3.25
Vasculitis	1.50 (6)	0	0	1.98
IgG4 related disease	0.50 (2)	0	0	1.44
Nephritis of anaphylactoid purpura	1.75 (7)	0	0	0.83
amyloidosis	2.01 (8)	0	0	1.13
light and heavy chain nephropathy	0.25 (1)	0	0	1.08
Nodular changes	0.25 (1)	0	0	0.41
Hepatitis B virus associated nephritis	2.26 (9)	0	0	0.69
Interstitial nephritis	2.00 (5)	20 (1)	0	0.79

## Discussion

As a degradation product of cross-linked fibrin, the presence of D-dimer indicates activation of the coagulation system. D-dimer has been become an important complementary tool for thrombotic disease diagnosis. The aim of our study was to investigate the association between the D-dimer level and the serious thrombotic complication after renal biopsy. To our knowledge, the present study is the first to examine whether correlations exist between D-dimer and thrombotic complications after renal biopsy. To get rid the effect of D-dimer level changes induced by surgery, we collected data before kidney biopsy, We indentified that elevated D-dimer, especially ≥2.00 μg/ml, was significantly associated with increased thrombotic complication after renal biopsy. Our finding motivates greater caution after native renal biopsies, especially in those with D-dimer ≥2.00 μg/ml measured before biopsy was performed. Perhaps these patients should be more closely observed after renal biopsies and may be use anticoagulants earlier.

Previous studies reported complications of renal biopies as gross hematuria, perinephric hematoma, a hemodynamically significant arteriovenous fistula, any radiological or surgical intervention^3, 17, 18^. They reported an incidence of major complications in the range of 6–8%^1, 2, 19^. Recent studies using realtime ultrasound and automated biopsy needles reported major complication rates of less than 5%^5, 18^. None of these studies connected thrombotic disease with renal biopies. The pathophysiology for the thrombophilic state in kidney disease is not fully understood. Regulatory coagulation proteins such as antithrombin III and protein S are oftern present in leakage that results from glomerular injury^20^. In response to protein loss into the urinary space, the liver compensates by increasing production of hemostatic proteins, especially factors I,VII,VIII,and X, shifting the hemostatic equilibrium in favor of pro-thrombosis^21, 22^. Elevation of platelet count has been regularly noted in patients with nephrotic syndrome, likely contributing to thrombotic risk. It has been shown that fibrin clot structure in nephrotic plasma is less porous than typical thrombi, possibly leading to increased resistance to fibrinolysis^23^. In our study, the higher FDP and the lower level AT-III in the group D-dimer levels ≥2.00 μg/ml which with higher rates of thrombotic complications also contribute to the hypercoagulable of patients with higher D-dimer level. The protein C pathway provides multiple important functions that regulate both haemostasis and host defence system in response to vascular injury. The anticoagulant protein C pathway is designed to regulate coagulation, maintain the fluidity of blood within the vasculature, and prevent thrombosis^24, 25^. The higher level of protein C in D-dimer ≥2.00 μg/ml group reflects the activated of anticoagulation system. Before and after the renal biopsy procedure the routine is stop any anti-platelet agents or anticoagulants and after the procedure the patient should lie flat on strict bed for 4 to 24 hours to reduce the bleeding complication, which could increase the risks of thrombotic complications in some patients. The renal biopsy procedure itself also stimulate the coagulation system.

D-dimer tests have been used in the evaluation of suspected thrombtic disorders for more than 20 years^10, 13^. D-dimer are typically elevated in patients with acute vein thromboembolism, myocardial infarction, stroke and other prothrombotic conditions^16, 26–28^. D-dimer is a sensitive but nonspecific marker for venous thrombosis. Recent guidelines recommend the use of initial D-dimer testing when evaluating patients with either a low or moderate pretest probability of DVT or PE^29^. If the D-dimer assay result is negative, DVT or PE is excluded and no further testing is necessary.

Levels of D-dimer are not only elevated with acute VTE, but also present in a wide variety of inflammatory conditions and connective tissue disorders, which contributes to the high level of D-dimer in lupus nephritis and crescentic nephritis. The main mechanisms of the coagulation derangement during systemic inflammatory activity are tissue factor-mediated thrombin generation and an imbalance of dysfunction of the normal physiologic anticoagulant mechanisms, such as the antithrombin system and the protein C system^30^. Inflammation and coagulation often interact. Inflammation activates coagulation, and vice versa, coagulation also modulates inflammatory activity. D-dimer as an end product of fibrinolysis, can promote the inflammatory cascade by activating neutrophils and monocytes, which induces inflammatory cytokines secretion and promotes hepatic synthesis of acute-phase proteins^31^. In addition to its diagnostic value, D-dimer levels are also of great prognostic significance and are associated with outcomes in many diseases. Serum D-dimer levels are associated with outcomes in patient with stroke, acute myocardial infarction, A acute aortic dissection, carcinoma, COPD^32–35^.

We do recognize that our study has several limitations. Firstly, this is a single-center study performed in a limited number of subjects of Chinese ethnicity. Only patients received renal biopsy between May 1, 2016 and September 30, 2016 were enrolled in this study. It might be insufficient to draw the same conclusion in other populations. Secondly, the different pathogenesis of kidney diseases as well as the therapeutics can affect the coagulation system. Thirdly, the incidence of thrombotic events in the whole patients after renal biopsy is relatively low. It could be attributed to the early anti-coagulant treatment on severe hypoalbuminemia. In light of its apparent clinical benefits in kidney disease, however, it will be viewed as unethical to conduct control trials in patients with uncontrolled hypercoagulation. Lastly, in an open-label study, our data may be subject to observer and selection bias as well. We acknowledge that a well-designed randomized, controlled trial could be more convincing to determine the diagnosis value of serum D-dimer in thrombotic complications.

In this study, we demonstrate that high serum D-dimer is associated with increased risk of thrombotic events in patients with renal biopsy. Our findings also indicate that serum D-dimer is a potential predictor for the incidence of venous thrombosis in kidney disease. Further cautions should be given to these subjects including consideration of early anticoagulant therapy after renal biopsy. It is of clinical significance to further determine if the pattern of serum D-dimer can link to the thrombotic complications in acute and chronic kidney diseases.
